# Previous hospital admissions and disease severity predict the use of antipsychotic combination treatment in patients with schizophrenia

**DOI:** 10.1186/1471-244X-11-126

**Published:** 2011-08-03

**Authors:** Albert Bolstad, Ole A Andreassen, Jan I Røssberg, Ingrid Agartz, Ingrid Melle, Lars Tanum

**Affiliations:** 1 Department of Psychiatry Research, Diakonhjemmet Hospital, P.O. Box 85 Vinderen, Oslo 0319, Norway; 2 Section of Psychosis Research, Clinic of Mental Health and Addiction, Oslo University Hospital, Ullevål Hospital, P.O. Box 4956 Nydalen, Oslo 0424, Norway; 3 Institute of Clinical Medicine, University of Oslo, P.O. Box 1171 Blindern, Oslo 0318, Norway; 4 Department of Psychiatric Research and Development, Akershus University Hospital and University of Oslo, Lørenskog 1478, Norway

## Abstract

**Background:**

Although not recommended in treatment guidelines, previous studies have shown a frequent use of more than one antipsychotic agent among patients with schizophrenia. The main aims of the present study were to explore the antipsychotic treatment regimen among patients with schizophrenia in a catchment area-based sample and to investigate clinical characteristics associated with antipsychotic combination treatment.

**Methods:**

The study included 329 patients diagnosed with schizophrenia using antipsychotic medication. Patients were recruited from all psychiatric hospitals in Oslo. Diagnoses were obtained by use of the Structured Clinical Interview for DSM-IV Axis I disorders (SCID-I). Additionally, Global Assessment of Functioning (GAF), Positive and Negative Syndrome Scale (PANSS) and number of hospitalisations and pharmacological treatment were assessed.

**Results:**

Multiple hospital admissions, low GAF scores and high PANSS scores, were significantly associated with the prescription of combination treatment with two or more antipsychotics. The use of combination treatment increased significantly from the second hospital admission. Combination therapy was not significantly associated with age or gender. Regression models confirmed that an increasing number of hospital admission was the strongest predictor of the use of two or more antipsychotics.

**Conclusions:**

Previous hospital admissions and disease severity measured by high PANSS scores and low GAF scores, predict the use of antipsychotic combination treatment in patients with schizophrenia. Future studies should further explore the use of antipsychotic drug treatment in clinical practice and partly based on such data establish more robust treatment guidelines for patients with persistently high symptom load.

## Background

Evidence-based recommendations and guidelines for psychopharmacological treatment of schizophrenia are regarded as important tools to improve the quality of clinical treatment, to guide decision-making and to reduce inappropriate variations in clinical practice [1]. The guidelines also intended to offer a more rational treatment strategy and thereby optimizing the therapeutic efficacy and reduce the side effects [2-4]. Even so, prescriptions of antipsychotics have often deviated from expert recommendations [1,5], and treatment guidelines are not implemented for all schizophrenia patients [6,7]. Since up to 90% of patients with schizophrenia may be excluded from clinical trials, there will naturally be a gap between clinical practice and study reports on the efficacy of treatment [8]. The variation in prescription patterns across different regions and countries [1,9,10] could also be due to differences in therapeutic traditions and treatment settings, attitudes towards antipsychotic drugs or cultural factors among psychiatrists.

It has been proposed that antipsychotic combination regimens are more frequently prescribed when the clinical course of the illness is complex or in treatment-resistant patients [10,11]. Studies of hospitalized patients with schizophrenia indicate a higher rate of antipsychotic combination treatment and use of First Generation Antipsychotics (FGA) compared to guideline recommendations [10,12,13]. Differences in prescription patterns could therefore be related to disease characteristics [1], although this has not yet been investigated in any catchment area sample representative of the majority of patients with schizophrenia.

The aim of this study was to explore the prescription pattern of antipsychotics among a sample of patients with schizophrenia receiving pharmacological treatment. The following research questions were addressed: 1) What types of antipsychotic medications are used in the treatment of schizophrenia in a Norwegian naturalistic catchment area sample? 2) Is the use of two or more antipsychotics associated with the severity of the disease as measured with the Global Assessment of Function (GAF), Positive and Negative Syndrome Scale (PANSS) and/or number of hospitalisations?

## Methods

### Sample

All the psychiatric hospitals in Oslo participate in the cross-sectional Thematically Organized Psychosis (TOP) Study, in which patients with psychotic disorders were recruited from outpatient as well as inpatient hospital units in a catchment area-based psychiatric service from 2003 to 2010. Up to 2008, we do not possess accurate data concerning out- or inpatient status at the time of inclusion in the study. A number of patients were recruited while being hospitalized, but actually included after discharge. The TOP study includes patients with a DSM-IV [14] diagnosis of schizophrenia and schizoaffective disorders, bipolar disorders and psychosis NOS. Patients were excluded if there was a previous history of head trauma, serious somatic illness or they were unable to give written, informed consent. For further information about the inclusion procedures, see [15-17]. A total of 329 patients, 213 (64.7%) men and 116 (35.3%) women, with schizophrenia fulfilled the inclusion criteria of the present study, including current use of antipsychotic medication and information on previous treatment history (For further patient characteristics, see Table 1).

**Table 1:** Background variables of patients included in the study (n = 329#)

	Mean (S.D)	Median (min. - max.)
Age (years), (n = 329)	31.9 (9.6)	30 (17-60)

Age of psychosis onset (years) (n = 140)	23.8 (8.3)	22 (7-54)

Duration of untreated psychosis (weeks) (n = 142))	134 (182)	76 (0-1040)

GAF-Symptom (n = 329)	40.0 (10.5)	38 (8-81)

GAF-Function (n = 329)	41.0 (9.5)	40 (21-81)

PANSS-positive (n = 305)	16.3 (5.9)	16 (7-32)

PANSS-negative (n = 307)	17.0 (6.6)	16 (7-43)

PANSS-general(n = 304)	33.3 (8.7)	33 (16-69)

PANSS-total (n = 302)	66.7(7.0)	67 (30-144)

Previous hospital admissions (n = 329)	3.5(5.3)	2 (0-40)

S.D.; standard deviation, GAF; Global Assessment of Functioning PANSS, Positive and Negative Syndrome Scale,

# A total of 329 patients, 213 (64.7%) men and 116 (35.3%) women, with schizophrenia were included. Among these 261 (79.3%) patients fulfilled the DSM-IV (16) criteria for the paranoid type of schizophrenia, 40 (12.2%) patients undifferentiated type, 16 (4.9%) disorganized type, 11(3.3%) residual type and 1 (0.3%) catatonic type.

The protocol was approved by The Norwegian Data Inspectorate and by the Regional Committee for Medical Research Ethics. All included patients received both written and oral information about the study and gave their written consent. The study was performed in full accordance with the Declaration of Helsinki (1965) and later revisions.

### Assessment

All patients included in the study went through the Structured Clinical Interview for DSM-IV Axis I disorders (SCID-I) [18] for diagnostic purposes. All raters were investigators in the study, with a background either as medical doctor or clinical psychologist. The raters were trained in clinical interviews and assessments including video and case report ratings. Tests for rating reliability were performed for the different diagnoses, PANSS and GAF scores. The diagnostic reliability was found to be satisfactory with an overall agreement for DSM-IV diagnostic categories of 82% with κ = 0.77 (95% CI: 0.60-0.94). The level of functioning was assessed by use of the split version of the Global Assessment of Functioning scale (GAF) [18]. The GAF rating was carried out by the investigators, with a satisfactory inter-rater reliability for GAF-F (ICC (1.1) = 0.86).

Relevant information on present and former drug treatment and previous hospitalisation were obtained from patient interviews and hospital records. If a patient used more than one antipsychotic drug at the time of the study, the drug prescribed in the highest dose, estimated from Defined Daily Doses (DDD), was defined as the primary therapeutic agent. If prescribed doses of two or more antipsychotics were equipotent, the medication with the longest duration was defined as the primary therapeutic agent. Secondary and tertiary therapeutic agents were established in line with this.

### Statistical analysis

Descriptive statistics was used for the initial analyses of frequencies, means and standard deviations (SD). Independent sample t-tests were used on between-group comparisons of means. Mann-Whitney test were used when skewed distribution of data were assumed. When dichotomous variables, we used Pearson Chi Square tests. Significant relationships were further explored by using a Backward Stepwise logistic regression model. Due to the low accuracy on the present inpatient versus outpatient treatment status, we did not include this factor in the logistic regression analyses. The effect sizes are presented as odds ratios. Nagelkerke's R-square was used to indicate goodness of fit. All analyses were performed using the Statistical Package for Social Sciences (SPSS), Version 14.

## Results

A total of 329 patients received antipsychotic pharmacological treatment as their primary therapeutic medication. Out of these, 305 (92.7%) patients received a second-generation antipsychotic (SGA) and 24 (7.3%) patients received a first generation antipsychotic (FGA). Olanzapine was the most frequent used primary medication (31.6%), followed by Quetiapine (17.6%), Aripiprazol (15.5%) and Risperidon (including Risperdal Consta) (12.4%), (See Table 2).

**Table 2:** Received primary therapeutic agent (PTA) in the study population, n = 329

Drug or treatment regimen	No. of patients (%)	Mean daily dose mg*	CPZ equivalents mg**
Second generation antipsychotic (SGA)	305 (92.7%)		

Olanzapine	104 (31.6%)	14.4	288

Risperidon or Risperdal Consta	41 (20.6%)	3.7	244

Quetiapine	58 (17.6%)	537,5	698

Aripiprazole	51 (15.5%)	14.2	189

Ziprasidon	20 (6.1%)	85.8	137

Amisulpride	11 (3.3%)	615.9	616

Clozapine	16 (4.8%)	390.6	391

Sertindole	4 (1.2%)	18.2	342

First generation antipsychotic (FGA)	24 (7.3%)		

Perphenazine or Perphenazine decan.	12 (3.6%)	8.9	111

Zuclopenthixol or Zuclopenthixol decan.	6 (2.1%)	20.0	80

Chlorprothixen	3 (0.9%)	116.7	233

Levomepromazine	1 (0,3%)	120	120

Chlorpromazine	1 (0.3%)	30.0	120

Flupentixol	1 (0.3%)	1.0	50

Haloperidol	0 (0%)		

No. of patients using only one antipsychotic drug	228 (69.3%)		

No. of patients using more than one antipsychotic drug	101 (30.7%)		

*Per oral medication only.

** CPZ equivalents; Chlorpromazine equivalents according to Kroken et al 2009: http://www.biomedcentral.com/1471-244X/9/24/table/T1

A total of 101 (30.7%) patients used two or more antipsychotics in combination. FGAs were used somewhat less frequent than SGAs (39 vs. 63 cases) as a second antipsychotic. Only 12 (3.2%) of the patients used three or more antipsychotics. The combination of SGA + SGA was used in 61 patients, SGA+ FGA in 37 patients, FGA+ FGA in only 2 patients and FGA + SGA were not used by any patient in this sample.

Patients using two or more antipsychotics scored on average significant lower on GAF-function and GAF-symptoms, while they scored significantly (p < 0.05) higher on PANSS-positive symptom scale and PANSS- negative symptom scale. However, they did not score significantly higher on the PANSS-general symptom scale (p = 0.056) and there was no association with age (See Table 3 and 4).

**Table 3:** Group wise comparing of means

	Patients with one antipsychotic	Patients with two or more antipsychotics
	Mean	Mean

Age (n = 329)	32.12	31.32

Age at onset of psychosis (n = 140)	24.11	23.06

Duration (weeks) of untreated psychosis (n = 142)	151	91

GAF-S (n = 329)	41.51	36.65

GAF-F (n = 329)	42.32	38.15

PANSS-positive (n = 305)	15.81	17.38

PANSS negative (n = 307)	16.16	18.82

PANSS general (n = 304)	32.75	34.82

PANSS total (n = 302)	64.65	71.29

Number of previous admissions(n = 329)	2.916	4.881

GAF-S; Global Assessment of Functioning,-Symptom, GAF-F Global Assessment of Functioning, - Functions, PANSS; Positive and Negative Syndrome Scale

**Table 4:** Independent sample t-test comparing group of patients with one antipsychotic versus patients with two or more antipsychotics

	T-test for equality of means		
	**t**	**Sig (2-tailed)**	**95% Confidence for the difference**
			**Lower - Upper**

Age (n = 329)	0.689	0.486	-1.457 - 3.060

Age at onset of psychosis (n = 140)	0.795*	0.429	-1.583 - 3.692

Duration (weeks) of untreated psychosis (n = 142)	2.318*	0.022	8.757 - 110.567

GAF-S (n = 329)	3.971	0.000	2.452 - 7.267

GAF-F (n = 329)	3.734	0.000	1.972 - 3.363

PANSS-positive (n = 305)	-2.187	0.030	-2.997 - -0.158

PANSS negative (n = 307)	-3.297	0.001	-4.239 - -1.070

PANSS general (n = 304)	-1.916	0.056	-4.195 - -0.056

PANSS total (n = 302)	-3.133	0.002	-10.821 - -2.471

Number of previous admissions (n = 329)	-2.859*	0.005	- 3.323 - -0.606

No. of previous admission dichotomized, (0 or 1 vs. 2 or more previous admissions)	-3.189*	0.002	-0.284 - -0.067

* Equal variances not assumed due to Levene's Test for Equality of Variances, p < 0.05

Number of previous admissions in the group of patients using only one antipsychotic drug was significantly lower than in the group of patients using two or more antipsychotic drugs (Mann-Whitney *U *= 8482,50, *Z *= -3.861, p-value = 0.000, r = -0.2129). Duration of untreated psychosis (DUP) did not differ significantly between the groups (Mann-Whitney *U *= 1790.00, *Z *= -1.134, *p *value = 0.257, r = -0.0951).

Pearson Chi-Square showed a significant relationship between two or more previous admissions and antipsychotic combination treatment (Value 9.086; Asymp. p-value = 0.003).

However, Pearson Chi-Square did not show any significant relationship between gender and antipsychotic combination treatment (Value 0.009; Asymp. p-value = 0.922). We have information on inpatient versus outpatient status in only 161 out of 329 patients. Only 12 (16.7%) out of 72 outpatients received antipsychotic combination treatment while 33 (37.1%) out of 89 inpatients at the time of inclusion received antipsychotic combination treatment. We found a significant relationship between inpatient status and antipsychotic combination treatment (Pearson Chi-Square Value 9.045; Asymp. p-value = 0.003).

As displayed in Table 5, the probability of two or more antipsychotics being used increased with the number of previous admissions to a psychiatric hospital. Among patients with two or more previous admissions, 36.8% received combination treatment. In contrast, only 21.9% of the patients with one or no previous admissions, and only 18.4% of patients not previously admitted, received such treatment. There was a nominal increase in patients receiving two or more antipsychotics up to the fourth previous admission. However, the portion of patients receiving two or more antipsychotics did not further increase with higher numbers of previous admissions. We used a stepwise backward logistic regression model exploring the individual strength of the possible predictors. We entered GAF-symptom, GAF-function, PANSS-positive, PANSS-negative, PANSS-general sub score and previous admissions into the regression model. The number of previous admissions was dichotomized into two groups, less than two admissions versus two or more admissions. Two or more previous hospital admissions appeared to be the far strongest predictor of combination treatment, followed by a low GAF symptoms score and a high PANSS-negative symptoms score (Table 6) The group of patients with two or more previous admissions showed an odds ratio of 2.445, for receiving two or more antipsychotics compared to patients with no or one previous admissions. Nagelkerkes R Square was 0.135 for the last step.

**Table 5:** Number of previous hospital admissions; comparison of patients with only one antipsychotic versus patients with two or more antipsychotics

		No. of previous admissions to psychiatric ward								
		0	1	2	3	4	5	6	7	8+

	N = 329	49	79	63	39	29	14	13	13	30

One antipsychotic	228 (69.3%)	40 (81.6%)	61 (77.2%)	46 (73.0%)	28 (71,8%)	13 (44.8%)	8 (57.1%)	8 (44.8%)	8 (61.5%)	16 (53.3%)

Two or more antipsychotics	91 (30.7%)	9 (18.4%)	18 (22.8%)	17 (27.0%)	11 (28.2.%)	16 (55.2%)	6 (42.9%)	5 (38.5%)	5 (38.5%)	14 (46.7%)

**Table 6:** Logistic regression model; Backward Stepwise (Wald) Previous admissions dichotomized 0-1 versus 2 or more

Patient factor	Odds Ratio (Exp.(B))	95.0% C.I. for OR	P-value	Wald
		Lower - Upper		
Step 4 (last step) *				

GAF-Symptom	0.953	0.924 - 0.983	0.002	9.239

PANSS-negative symptoms	1,048	1.007 - 1.090	0.022	5.242

Two or more hospital admissions	2.445	1.416 - 4.225	0.001	10.258

GAF; Global Assessment of Functioning, PANSS; Positive and Negative Syndrome Scale, OR; Odds Ratio

*Nagelkerke R Square = 0.135

## Discussion

The main finding of this study is that the prevalence of antipsychotic combination treatment increased with number of hospital admissions, severity of the disease as measured with PANSS, and level of dysfunction, as measured with GAF. The current finding that previous hospital admissions were related to antipsychotic combination treatment is in line with Kroken el. al [9] who found that in-patient treatment in the previous 12 months predicted polypharmacy. As seen from table 5, increase in number of hospital admissions beyond four did not seem to increase to probability of receiving two or more antipsychotics. We dichotomized the sample and chose a cut-off between one and two previous admissions. The reason for choosing this cut-off value was primarily the clinical relevant distinction between patients who were readmitted and those who were not. In a number of the patients first admittance to hospital was not necessarily due to problems with ongoing treatment, but due to a more acute or dramatic onset of symptoms of schizophrenia. Choosing a cut-off between one and two admissions therefore reflects to a greater extend patients with poor compliance or lack of response to ongoing treatment.

The cut-off points could just as well have been set between two and three, or between three and four previous admissions, providing slightly higher odds ratios. However, more than five previous admissions did not further increase the probability of receiving an antipsychotic combination treatment. Our results seem to be in line with studies that suggest a combination of antipsychotics as an option in non-responders with a higher degree of relapse, or in patients with more severe schizophrenia [5,12,19,20]. The finding that the use of antipsychotics were mainly in accordance with guidelines up to the second admission to hospital, supports the hypothesis that antipsychotic combination therapy is more likely to be prescribed when treatment according to guidelines has not achieved an adequate therapeutic response.

Previous studies have not reported any significant correlation between prescription pattern and decline in global- or daily functioning, measured with GAF [9,21]. This could be due to a type II error, at least in one of the studies, since this only included inpatients [9]. The current finding of PANSS score versus combination treatment, has not been reported earlier. A higher level of current psychotic symptoms, as measured with total PANSS scores, further supports the hypothesis that antipsychotic combination therapy is more likely to be prescribed when guideline treatments have not achieved an adequate therapeutic response.

Our naturalistic sample of patients consisting of both inpatients and outpatients at the time of the examination, might be more representative for the population of patients with schizophrenia at various stages of the illness, providing a relatively wide spectrum in symptom levels and functioning and thus GAF and PANSS scores. This probably enabled us to detect important associations that are difficult to find in more selected groups of patients e.g. inpatients only.

Duration of untreated psychosis (DUP) was verified in only a portion of the patients but did not show any significant relationship to combination treatment with antipsychotics. This may be in line with our finding that age did not show any significant relationship with such treatment either. Future studies should further explore the role of DUP with regard to medication regimens.

The overall rate of antipsychotic combination treatment among our patients was comparable to other naturalistic studies [9,12]. In our study SGAs were used more frequently as the preferred antipsychotic drug than reported from some European studies performed during the same time period [9,11,22,23], but was in line with other study reports [24]. The use of FGA as a primary therapeutic agent was relatively infrequent in our study. The variation in prescription patterns of SGAs may be attributed to both guideline adherence and how the public health systems work in different countries, including to what extent prescriptions of all antipsychotic medications are reimbursed by the social security program, as well as differences in the hospitals' financial schemes which influence the choice of low-versus high-cost medications.

Evidence-based guidelines for the psychopharmacological treatment of schizophrenia are important for securing a high quality of clinical practice including rational strategies to minimize adverse effects. However, the knowledge is rather scarce on how to guide treatment decisions in non-responders to antipsychotic monotherapy, which may be reflected by the lack of evidence-based recommendations for this group of schizophrenia patients. A better discrimination between subgroups of patients with different clinical courses of the illness is therefore needed when proposing new recommendations, moving today's guidelines with their "one size fits all" approach to antipsychotic medications closer to clinical practice.

The current body of evidence to support a combination of two or more antipsychotics in schizophrenia is not conclusive [20,24-26], even though antipsychotic combination treatment may be superior to monotherapy in a limited number of patients [12,26,27]. A few randomized controlled trials have reported treatment with clozapine in combination with a second antipsychotic, to be superior to clozapine in monotherapy in subgroups of patients [27].

The current study involved all psychiatric hospitals in Oslo and included both in- and outpatients. The public health care service in Norway is good and provides adequate treatment for all psychiatric patients. There is no privately financed health care that offers long-term treatment for patients with schizophrenia, which enabled us to collect representative data on current treatment with a rather low degree of selection bias.

## Limitations

Our data are based on a sample of cooperating patients who agreed to join the study, including all assessments and interviews. Many patients were outpatients, indicating a higher degree of treatment compliance compared to inpatients or long-term hospitalized patients. In contrast, studies that only recruit inpatients may have a selection bias towards more severe and treatment-refractory cases.

## Conclusions

Patients with previous hospital admissions and disease severity measured by high PANSS scores and low GAF scores were more likely to receive an antipsychotic combination treatment. Future studies should further explore the use of antipsychotic drug treatment in clinical practice and partly based on such data establish more robust treatment guidelines for patients with persistently high symptom load.

## Abbreviations

DDD: Defined Daily Doses; FGA: First Generation Antipsychotic; SGA: Second Generation Antipsychotics; GAF-F: Global Assessment of Functioning; SCID-I: The Structured Clinical Interview for DSM-IV; PANSS: Positive and Negative Syndrome Scale; DUP: Duration of Untreated Psychosis; TOP: Thematically Organized Psychosis;

## Competing interests

OAA and LT have received a speaker's honorarium from Astra-Zeneca, GlaxoSmithKline, Janssen-Cilag and Bristol Myers Squibb. LT has also received a speaker's honorarium from Sanofi-Aventis. IM have received a speaker's honorarium from Astra-Zeneca, Eli-Lilly, Janssen-Cilag and Lundbeck. IA is an unpaid consultant to Eli Lilly. No competing interests.

## Authors' contributions

AB: collecting data, analysis, drafting and revising the manuscript. OAA: conception of the study, collecting data, analysis, drafting and revising the manuscript. JIR: conception of the study and revising the manuscript. IA: conception of the study, collecting data and revising the manuscript. IM: conception of the study, collecting data and revising the manuscript. LT: conception of the study, analysis, drafting and revising the manuscript. All authors have read and approved the final manuscript.

## Pre-publication history

The pre-publication history for this paper can be accessed here:

http://www.biomedcentral.com/1471-244X/11/126/prepub
